# Tuning mitochondrial structure and function to criticality by fluctuation-driven mechanotransduction

**DOI:** 10.1038/s41598-019-57301-1

**Published:** 2020-01-15

**Authors:** Erzsébet Bartolák-Suki, Béla Suki

**Affiliations:** Department of Biomedical Engineering, Boston University, Boston, MA 02215 United States

**Keywords:** Phase transitions and critical phenomena, Biological physics, Energy metabolism

## Abstract

Cells in vascular walls are exposed to blood pressure variability (BPV)-induced cycle-by-cycle fluctuations in mechanical forces which vary considerably with pathology. For example, BPV is elevated in hypertension but reduced under anesthesia. We hypothesized that the extent of mechanical fluctuations applied to vascular smooth muscle cells (VSMCs) regulates mitochondrial network structure near the percolation transition, which also influences ATP and reactive oxygen species (ROS) production. We stretched VSMCs in culture with cycle-by-cycle variability in area strain ranging from no variability (0%), as in standard laboratory conditions, through abnormally small (6%) and physiological (25%) to pathologically high (50%) variability mimicking hypertension, superimposed on 0.1 mean area strain. To explore how oxidative stress and ATP-dependent metabolism affect mitochondria, experiments were repeated in the presence of hydrogen peroxide and AMP-PNP, an ATP analog and competitive inhibitor of ATPases. Physiological 25% variability maintained activated mitochondrial cluster structure at percolation with a power law distribution and exponent matching the theoretical value in 2 dimensions. The 25% variability also maximized ATP and minimized cellular and mitochondrial ROS production via selective control of fission and fusion proteins (mitofusins, OPA1 and DRP1) as well as through stretch-sensitive regulation of the ATP synthase and VDAC1, the channel that releases ATP into the cytosol. Furthermore, pathologically low or high variability moved mitochondria away from percolation which reduced the effectiveness of the electron transport chain by lowering ATP and increasing ROS productions. We conclude that normal BPV is required for maintaining optimal mitochondrial structure and function in VSMCs.

## Introduction

Intracellular structures form complex dynamic networks. Both the cytoskeletal actin^1,2^ and microtubule^3,4^ networks actively self-organize themselves into adaptable structures using ATP- and GTP-dependent processes, respectively. Mitochondria also form a dynamic network that constantly undergoes energy-dependent fission and fusion^5–7^, two processes that maintain the network in some optimal configuration for proper supply of various cell functions with ATP.

A model-based analysis of the mitochondrial network suggests that the connectivity of the mitochondria may undergo a percolation-like phase transition^8^ with at least one contiguous mitochondrial cluster spanning the cell, which allows rapid energy supply at virtually any intracellular location. Recent experiments also provided evidence that the balance between fission and fusion controls such transitions^9^. In response to localized oxidative stress, inter-mitochondrial communication is promoted by a cell-wide phase transition in mitochondrial depolarization when mitochondrial clusters with reactive oxygen species (ROS) beyond a critical threshold level tend to form a large percolation-like cluster^10^. These studies link mitochondrial function to the complexity and criticality of mitochondrial structure.

It is important to recognize that the above results were obtained in static cell cultures. However, most cells in the body are exposed to a dynamic mechanical environment due to body motion, muscle contraction, blood pressure (BP) and breathing. The cytoskeleton actively probes the extracellular matrix (ECM)^11–13^ and participates in mechanotransduction, the conversion of mechanical forces to signaling^14^. Since mitochondria strongly interact with the cytoskeletal networks^15–18^, it is not surprising that mechanical cues such as fluid flow-related shear stress^19^, mechanical stretch^20,21^ or ECM stiffness^22^ affect mitochondrial structure and function. Furthermore, the external mechanical environment is invariably noisy due for example to beat-to-beat blood pressure variability (BPV). Consequently, cells and their mitochondria also respond to fluctuations in mechanical factors, a process called fluctuation-driven mechanotransduction (FDM)^23^. Indeed, we recently reported that ATP production, monitored via the mitochondrial membrane potential, was maintained at a high level in vascular smooth muscle cells (VSMCs) when they were exposed to a variable-amplitude cyclic stretch pattern mimicking normal BPV compared with monotonous constant-amplitude cyclic stretch^24^.

It has been reported that BPV is elevated in patients with hypertension and autonomic failure when subjects were active^25^, but BPV is low in patients with autonomic failure during rest^25^ and in subjects under anesthesia^26^. Thus, the amount of fluctuations in mechanical forces that cells experience *in vivo* may vary considerably. Accordingly, we hypothesized that there is an optimal level of fluctuations in mechanical forces applied to VSMCs that keeps the mitochondrial network structure near the percolation transition which in turn also determines ATP and reactive oxygen species (ROS) production. To test this hypothesis, we exposed VSMCs in culture to gradually increasing cycle-by-cycle variability in stretch amplitude ranging from no variability, as in standard laboratory conditions, through pathologically small variability as in anesthesia to extremely high variability mimicking hypertension. To shed light on how oxidative stress and ATP-dependent processes interact with the ability of FDM to organize mitochondrial structure near criticality, the experiments were repeated in the presence of hydrogen peroxide (H_2_O_2_) and AMP-PNP, an ATP analog and competitive intracellular inhibitor of ATPases^27,28^. Since AMP-PNP is non-hydrolysable, treatment of cells with AMP-PNP can be used to test the extent to which FDM requires ATP hydrolysis.

## Results

### Variability in strain affects mitochondrial structure

**Figure 1:**
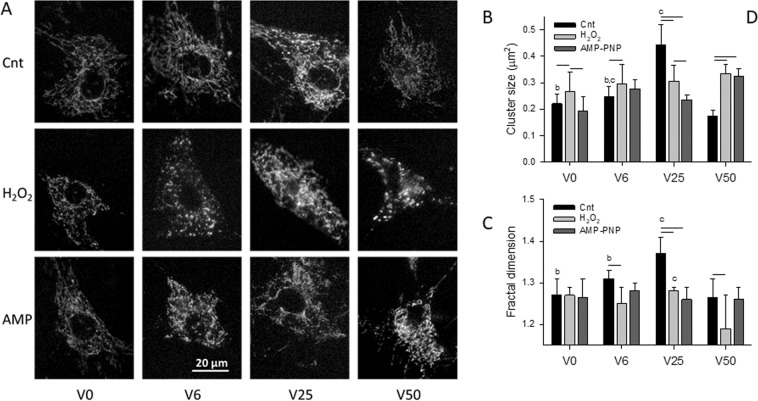
(**A**) Example images of the mitochondrial network in vascular smooth muscle cells (VSMCs) cultured on elastic membranes. Mitochondria were labeled with tetramethylrhodamine methyl ester (TMRM) and images were taken after 4 hours of equi-biaxial stretch with a mean peak strain amplitude of 0.1 and superimposed cycle-by-cycle variabilities of 0%, 6%, 25% and 50% denoted respectively by V0, V6, V25 and V50 in the absence (Cnt: control) or presence of the ATP analogue and ATPase inhibitor AMP-PNP (AMP) or hydrogen peroxide (H_2_O_2_) to induce oxidative stress. Means and SDs of the mean cluster within a cell (**B**) and the fractal dimension D_f_ of the entire mitochondria per cell (**C**). Both structural parameters were obtained under V0, V6, V25 and V50 in the absence (Cnt) or presence of AMP or H_2_O_2_. Two-way ANOVA was used to analyze the data. Bars above two groups within a given variability condition denote statistically significance. The letters a, b, and c above bars denote statistically significant difference relative to V6, V25 and V50, respectively.

Live cell imaging of VSMCs was carried out to visualize active mitochondria labeled with the mitochondrial membrane potential sensor dye tetramethylrhodamine methyl ester (TMRM). Example images of the mitochondrial network are shown in Fig. 1A as a function of cycle-by-cycle strain variability (V0, V6, V25 and V50 representing 0%, 6.25%, 25% and 50% variability, respectively) superimposed on peak area strain of 0.1 in the presence and absence of AMP-PNP, an inhibitor of ATPases, and hydrogen peroxide (H_2_O_2_) which mimics the conditions of oxidative stress. After thresholding the images, the mitochondrial structure was first characterized by the average cluster size per cell (Fig. 1B) which was the largest under V25 in control cells and the smallest under V50 which did not differ from V0. H_2_O_2_ exposure increased the cluster size for all variabilities except V25, whereas AMP-PNP reduced and increased cluster size relative to control at V25 and V50, respectively. Alternatively, both H_2_O_2_ and AMP-PNP eliminated any dependence of cluster size on strain variability. As a second level of structural analysis, we computed the fractal dimension D_f_ (Fig. 1C) representing the overall space filling capacity and complexity of mitochondrial network. D_f_ showed a maximum during V25 in control cells; however, H_2_O_2_ and AMP-PNP eliminated the dependence of D_f_ on variability except between V25 and V50 during H_2_O_2_.

### Physiological strain variability leads to mitochondrial percolation

**Figure 2:**
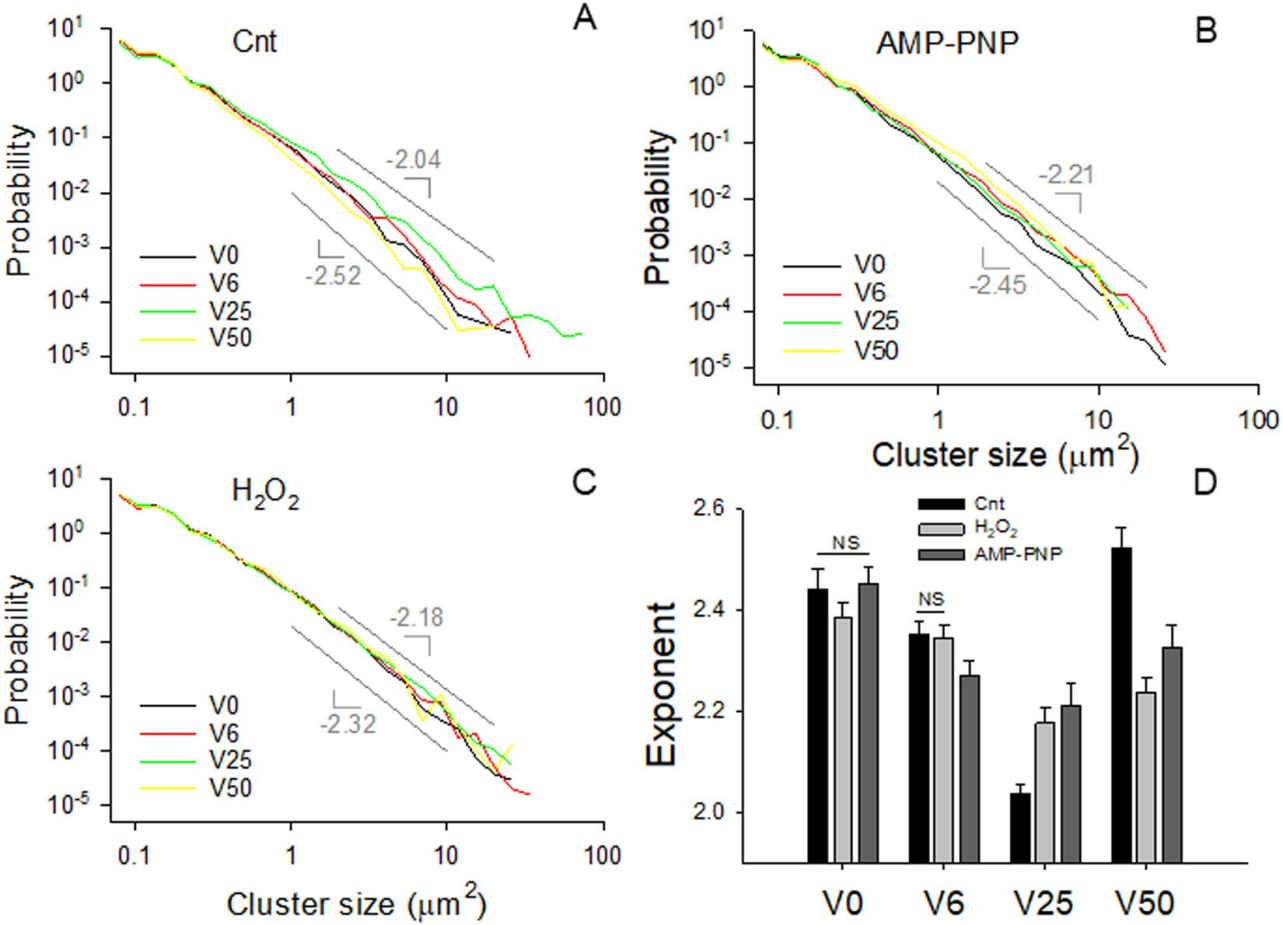
Power law probability density distributions of activated mitochondrial cluster sizes including all clusters from all cells in control (**A**) and during AMP-PNP (**B**) and H_2_O_2_ (**C**) treatments. The distributions were calculated for each stretch pattern. The smallest and largest slopes are indicated by the gray lines. The statistics of the corresponding exponents (negative of slopes) are summarized in panel D. All exponents are different except where NS is indicated.

To assess whether strain fluctuations influenced the connectivity of the activated mitochondrial network, we computed the probability density distributions of cluster sizes. Under all conditions, the distributions were well described by power laws, defined as a linear decrease on double logarithmic graphs, over about 2 orders of cluster sizes (Fig. 2A–C). The corresponding exponents (Fig. 2D), defined as the negative slope of a straight line fit of the data on a log-log graph, ranged between 2.05 and 2.5. For control cells, the exponent exhibited a minimum at V25 with a value very of 2.038 ± 0.016 which was not significantly different from the known percolation exponent of 2.05 in 2D^29^. While the exponents varied less with strain variability during H_2_O_2_ and AMP-PNP exposure, they still showed a minimum at V25 with values that were significantly higher than those in control cells.

**Figure 3:**
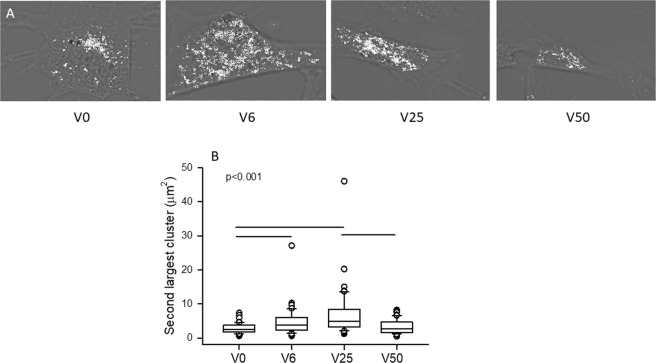
(**A**) Gray scale phase contrast images of VSMCs following 4 hours of stretch with V0, V6, V25 or V50. The mitochondrial clusters obtained by thresholding the TMRM images are superimposed in white to visualized cluster fragmentation and possible percolation. (**B**) Box plots of the size of the second largest cluster. The line in the box indicates the median whereas the ends of the boxes define the 25th and 75th percentiles, with the error bars defining the 10th and 90th percentiles. All conditions are statistically significantly different except V6 is not different from V25 and V50.

**Figure 4:**
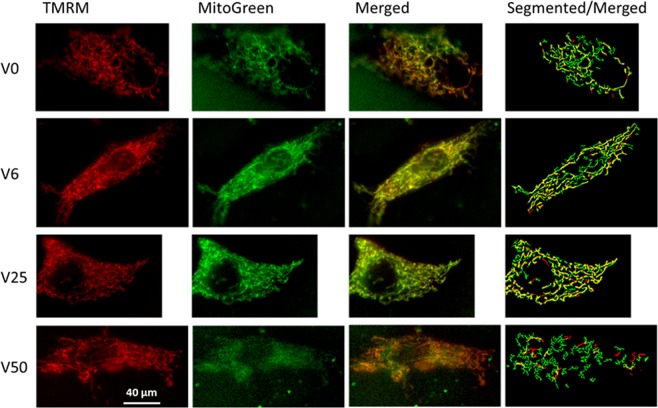
Example images of cells co-labeled with TMRM (column 1, red) and MitoTracker Green (column 2, green) following stretching with V0, V6, V25 and V50. The third column shows the merged images from columns 1 and 2 with the co-labeled mitochondria appearing in yellow. Column 4 demonstrates overlaid segmented images of columns 1 and 2 with yellow showing pixels that had both TMRM and MitoTracker Green. Segmentation was obtained using a fibermetric algorithm (see Methods). The TMRM aligns reasonably well with the MitoTracker Green. There are occasional red pixels without green which are an artifact of labeling and/or thresholding. Also note that the networks are more fragmented under V0 and V50 than under V6 and V25.

The increased mean cluster size, D_f_ and especially the value of the exponent during V25 in control cells suggest that percolation has been reached by the activated mitochondria. This possibility was further examined by superimposing the clusters on phase contrast images of control cells (Fig. 3A). The clusters appear small and relatively isolated in the case of V0 whereas progressively denser mitochondrial clusters are seen for V6 and V25. However, at V50, mitochondrial cluster structure appears substantially more fragmented than for V25. Note that for V25 and even for V6, clusters seem to spread through the cell indicating percolation. To verify this, we determined the size of the second largest cluster in each cell which is known to exhibit a maximum at the percolation threshold^30^. As Fig. 3B demonstrates, the second largest cluster is indeed maximized by V25 while V50 does not differ from V0 and V6. To examine the spatial organization of activated mitochondria relative to all mitochondria, we also visualized the mitochondrial clusters co-labeled with TMRM and MitoTracker Green which localizes to mitochondria regardless of the membrane potential (Fig. 4). Generally, activated mitochondria were well aligned with the total mitochondria in all conditions (except V50) and showed tubular structures especially under V6 and V25. It is also apparent that at V0 and especially at V50, the segmented images showed more mitochondrial fragmentation than at V6 and V25 suggesting the lack of percolation of all mitochondria at V0 and V50. In the specific examples of the segmented images showing the superposition of TMRM and MitoTracker Green (Fig. 4 right column), the activated mitochondria occupied 32%, 49%, 58% and 23% of the total mitochondria for V0, V6, V25 and V50, respectively.

### Effects of strain variability on mitochondrial proteins

**Figure 5:**
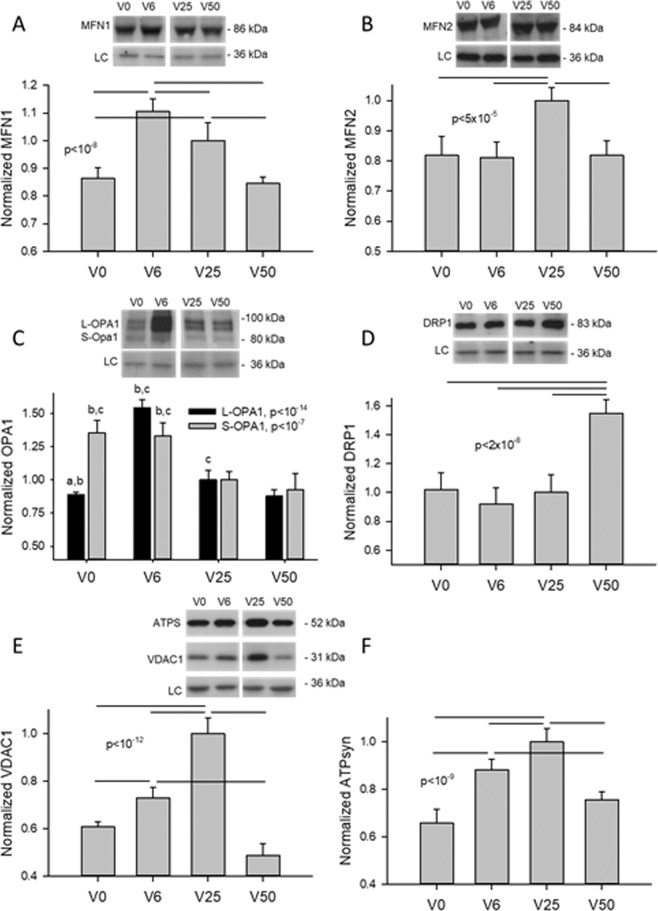
Example western blots and statistical analyses of mitofusins (MFN1 and MFN2), OPA1, DRP1, VDAC1 and β subunit of the ATP synthase as a function of strain variability (V0, V6, V25, and V50). GAPDH was used as loading controls. Data are expressed as means and SDs. Bars above the data represent statistically significant differences. In panel C, the letters a, b and c represent statistically significant differences compared to V6, V25 and V50, respectively. LC: loading control.

To shed light on the underlying molecular mechanisms, we quantified the levels of mitofusin-1 (MFN1) and mitofusin-2 (MFN2), which are required for outer mitochondrial membrane fusion^31^, both the short (S) and long (L) forms of the dynamin-related GTPase protein Optic Atrophy 1 (S-OPA1 and L-OPA1), which are required for inner membrane fusion^32^, and the dynamin-related protein 1 (DRP1), which regulates mitochondrial fission^33^. As Fig. 5 (panels A–D) demonstrates, the fusion promoting proteins MFN1 and L-OPA1 showed a peak at V6 followed by a decrease at V25, V0 and V50 whereas MFN2 had a single peak at V25. Interestingly, S-OPA1, which contributes to fission^34^, was upregulated by V0 and V6. DRP1 did not depend on strain variability between V0 and V25, but it was significantly higher at V50 compared to the rest explaining the fragmented network seen in Fig. 4 (bottom right image). Additionally, the expressions of the voltage-dependent anion channel 1 (VDAC1), which facilitates ATP transport to the cytosol^35^, as well as the β subunit of the ATP synthase also showed a distinct peak at V25 (Fig. 5E,F, respectively).

### Functional consequences of mitochondrial percolation

**Figure 6:**
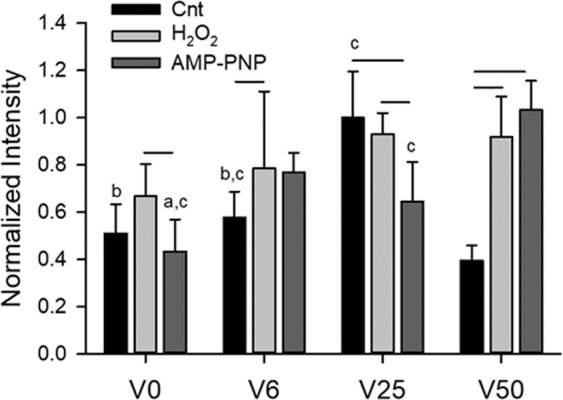
Means and standard deviations (SD) of the TMRM intensity of approximately 100 cells in each group. Two-way ANOVA was used to analyze the data. Bars above two groups within a given variability condition denote statistically significance. The letters a, b, and c above bars denote statistically significant difference relative to V6, V25 and V50, respectively.

The functional consequences of the variable stretch-induced re-organization of the mitochondrial structure were examined by determining the levels of TMRM intensity, representing mitochondrial membrane potential^36^ as a measure of ATP production rate^37^, the levels of mitochondrial and whole cell ROS as well as total cellular ATP amount. Quantitative analysis of TMRM intensity (Fig. 6) demonstrates that in the control group, ATP production rate exhibits a peak at V25 dropping to the lowest level at V50 which is different from V6 but not V0. Interestingly, H_2_O_2_ eliminated any dependence of intensity on variability, which was mostly due to the increase in intensity in all conditions except at V25. Unexpectedly, however, following AMP-PNP, the maximum intensity is seen at V50, which was much larger than that in control and also significantly different from all groups (except V6).

**Figure 7:**
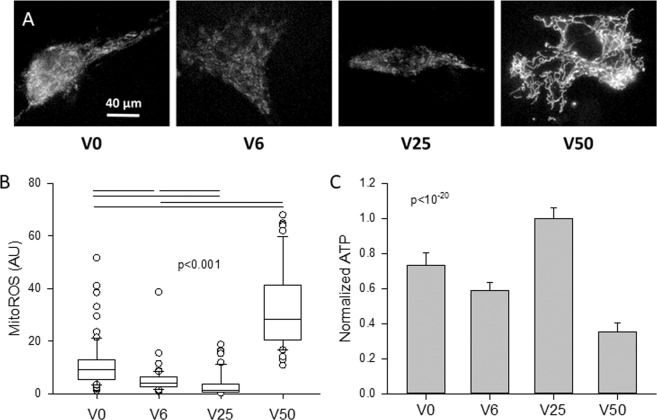
Example images (**A**) and statistics (**B**) of mitochondrial ROS levels after VSMCs were stretched with V0, V6, V25 or V50 and labeled with MitoSOX™ Red mitochondrial superoxide indicator. Approximately 50 cells per group were included in the statistics. The medians are statistically different between all conditions. (**C**) Means and SDs of total ATP normalized to unity under V25. All conditions are statistically significantly different.

**Figure 8:**
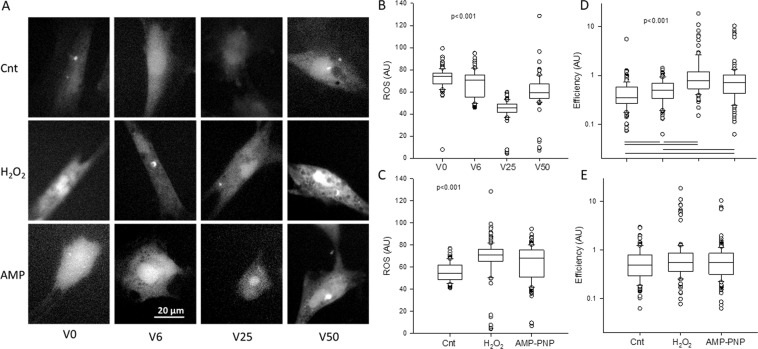
(**A**) Example images of cellular reactive oxygen species (ROS) measure after VSMCs were stretched with V0, V6, V25 or V50 in the absence or presence of AMP-PNP (AMP) or hydrogen peroxide (H_2_O_2_). (**B**) Box plots of ROS following the 4 different stretching patterns. For each variability, data were pulled from the control, AMP-PNP as well as H_2_O_2_ conditions. The medians are statistically different between any conditions. (**C**) Box plots of ROS as a function of treatment with all variabilities pulled for each treatment. All groups are statistically different from each other. (**D**) Box plots of efficiency defined as the ratio of mean TMRM intensity and mean ROS intensity in each VSMC following the 4 different stretching patterns. For each variability, data were pulled from the control, AMP-PNP as well as H_2_O_2_ conditions. The statistically significant differences between conditions are shown by bars under the data. (**E**) Box plots of efficiency as a function of treatment with all variabilities pulled for each treatment. The medians are not statistically significantly different.

Example images of mitochondrial ROS are shown in Fig. 7A and the corresponding intensities are summarized in Fig. 7B. The ROS levels, labeled with MitoSOX Red mitochondrial superoxide indicator, were significantly different from each other reaching a minimum at V25 and a maximum at V50. Interestingly, total ATP amount in cells also has a maximum at V25 followed by V0, V6 and then V50 (Fig. 7C). Whole cell ROS images are displayed in Fig. 8A together with their statistics in Fig. 8B,C. Independent of H_2_O_2_ and AMP-PNP, a minimum was seen at V25 whereas independent of strain variability, both H_2_O_2_ and AMP-PNP raised cellular ROS levels above those in control cells. To examine the relation between ROS and ATP production, we computed a metric of cell metabolic efficiency, defined as the ratio of the mean TMRM and the mean ROS per cell (Fig. 8D,E). The efficiency was the highest at V25 independent of H_2_O_2_ and AMP-PNP treatments. Alternatively, when data were grouped for all variabilities in control, H_2_O_2_ and AMP-PNP, no difference was seen in efficiency.

## Discussion

The primary findings of this study are that the amount of cycle-by-cycle fluctuations in peak area strain acts as a strong regulator of the mitochondrial network structure. Alterations in network complexity were also accompanied by changes in key regulatory proteins as well as ATP and ROS production in the mitochondria of control VSMCs. This suggests that in these cells, fluctuation-driven mechanotransduction (FDM) regulates mitochondrial bioenergetics. The underlying processes, however, are reduced or inhibited by oxidative stress and metabolic disturbance.

Following stretching of VSMCs with different amounts of strain variability, mean cluster size (Fig. 1B) as well as the network’s fractal dimension D_f_ (Fig. 1C) during V6 and V50 were reduced to levels comparable to those during V0 despite significant differential regulation of fusion and fission proteins (Fig. 5). To explain these results, we first consider the amount of mechanical energy communicated to the cell during stretching. Assuming that cells can be considered a simple linearly elastic material, we have shown that the energy during variable stretching is related to the fluctuations in cycle-by-cycle amplitude^23^. The energy density during variable stretch (*E*_*VS*_) can be written in the following form:

1 $${E}_{VS}={E}_{V0}+\frac{1}{2}Y{\sigma }^{2}$$

where VS is one of the variable stretch patterns (V6, V25 or V50), ${E}_{V0}$ is the energy during V0, *Y* is the stiffness of the cell and *σ*^2^ is the variance of the fluctuations in peak strain amplitude corresponding to 6, 25 or 50% variability. Thus, stretching cells with more strain variability increases the elastic energy communicated to the cell. Part of this energy is dissipated due to stretching the viscoelastic cytoskeleton^38^, deforming the mitochondrial network^39^ and upregulating mitochondrial and other proteins. The remaining energy may be converted into re-organizing and building a more complex mitochondrial network.

Network organization is a function of the availability of the fission-fusion regulatory proteins. While MFN1 (Fig. 5A) and L-OPA1 (Fig. 5C) were elevated following V6, any increase in cluster size and D_f_ (Fig. 1) was counter balanced by the high level of S-OPA1 (Fig. 5C) which promotes fragmentation^34^. The high level of MFN1 at V6 can be protective against mitophagy by tubular network formation (Fig. 5). On the other hand, the high level of MFN2 at V25 (Fig. 5F) can induce the expression of mitochondrial complexes I, IV and V through which it energizes mitochondrial metabolism^40^. Since V25 also induces the expression peroxisome proliferator-activated receptor γ coactivator (PGC-1α)^24^, a master regulator of mitochondrial biogenesis^41^, V25 has the potential to re-organize the network by enhancing fusion as well as adding new mitochondria to the existing network. These fundamental biochemical processes can in turn lead to an increase in network complexity (Fig. 1B,C).

The increased complexity of the activated mitochondria at V25 is also reflected in the fact that the exponent of the power law distribution has a minimum (Fig. 2D) and D_f_ has a maximum (Fig. 1C) suggesting that the largest clusters should occur during V25 in agreement with the cluster sizes (Fig. 1B). Interestingly, the value of the exponent is 2.05 in accord with percolation in 2D^29^ which can also be seen from the cluster structure within the cell (Fig. 3A) that maximizes the second largest cluster (Fig. 3B), a signature of percolation^30^. These results provide evidence that the active mitochondria reached percolation at V25. Indeed, percolation has been suggested to occur in mitochondrial networks^8–10^. However, we find that in VSMCs which are exposed to BPV-induced fluctuations in stretch *in vitro*, percolation of activated mitochondria only occurs during the dynamic conditions provided by V25. Since the activated mitochondria constitute a subset of all mitochondria, the entire mitochondrial network must also percolate; indeed, the activated clusters were best aligned with and occupied the largest fraction of all mitochondria under V25 (Fig. 5). Because the network undergoes continuous fission and fusion, the non-equilibrium steady state cluster sizes and their distribution are likely a result of reversible cluster-cluster aggregation^42,43^ rather than random percolation. The random bond breaking in cluster-cluster aggregation alters the fractal dimension and introduces time dependent size distributions^43^. Interestingly, the corresponding value of D_f_ is ~1.47 in 2D^44^ which is not far from our value of 1.37 following V25 (Fig. 1C). Nevertheless, the exponents and D_f_ values under V0, V6 and V50 are not similar to known aggregation processes. A limitation of the reversible aggregation model is that it assumes random aggregation, breaking and diffusion. In real cells, mitochondria move along organized microtubules^45^ and both fission and fusion are tightly regulated GTP-dependent processes^46^. This might also explain why the exponent of the cluster distribution reaches the percolation value but D_f_ is lower than the value in random percolation. Consequently, prediction of the biological and structural responses of the mitochondria to stimulations such as oxidative stress and ATP deprivation is currently not possible with the reversible random aggregation kinetic models.

Equation 1 also suggests that cells during V50 received the largest amount of elastic energy yet cluster size (Fig. 1B) as well as D_f_ (Fig. 1C) were lower during V50 than during V25. A potential explanation is that the largest strain fluctuations during V50 may become sufficiently high to induce fission or directly rupture mitochondria both of which dissipate energy. Indeed, various mechanical forces have been found to induce DRP1-dependent fission^47^. Furthermore, mitochondria have also been shown to rupture during slow stretch in lung fibroblasts^48^. Fragmentation of the network due to physical forces in turn upregulated total cellular DRP1 (Fig. 5D). Thus, the increasing fusion from V0 to V25 together with the breakdown of the network at V50 lead to an optimal mitochondrial structure with maximum complexity when strain variability was 25% during V25, which corresponds to normal BPV.

The optimal network complexity following V25 also allowed a larger part of the mechanical energy to be converted into chemical energy stored in ATP that was reflected in the TMRM intensity (Fig. 6) and ATP level (Fig. 7C). The underlying molecular mechanism at least in part is that FDM also maximized the expressions of the ATP synthase (Fig. 5F) directly responsible for ATP production and the VDAC1 channel (Fig. 5E) which allows the release of ATP into the cytosol^35^ (Fig. 5E). These findings are intriguing since the expressions of these proteins correlated strongly with the power law exponents in Fig. 2D with r^2^ values of 0.747 for the ATP synthase (not shown) and 0.982 for the VDAC1 (Fig. S1A). Furthermore, the power law exponent correlated even more strongly with TMRM intensity as a measure of ATP production rate (r^2^ = 0.995, Fig. S1B). These biochemical-structure-function relations signify a strong feedback between the production and release of ATP and the complexity of the mitochondrial network structure. It must also be pointed out, however, that part of the total cellular ATP is produced by glycolysis. For example, in cardiomyocytes after one day in culture, approximately 35% of ATP was derived from glycolysis^49^. Nevertheless, under baseline conditions, the mitochondrial oxidative phosphorylation dominates intracellular ATP generation^50^ and hence the FDM-induced changes in ATP (Fig. 7C) are likely a result of the increased mitochondrial ATP production rate (Fig. 6) and possibly alterations in consumption rates.

ATP production rate is primarily driven by the mitochondrial inner membrane potential^37^ which in turn is influenced by mitochondrial clustering governed by MFN2 which promotes the fusion of smaller clusters to form larger ones^51^. Alternatively, inhibiting DRP1 with silencing RNA enhances network formation and reduces ATP production whereas inhibition of oxidative phosphorylation induces network fragmentation and increases ROS production^52^. Therefore, due to the competing influences of fission and fusion, it may be expected that ATP production is maximized under the condition that also maximizes cluster size. Our data in Figs. 1B and 6 support this notion. However, the maximum cluster size was achieved due purely to the dynamic fluctuations in mechanical forces, a condition present *in vivo*, but not considered in past studies. In a recent study^24^, we reported that TMRM intensity, a marker of mitochondrial membrane potential^36^, increased approximately linearly with the logarithm of cluster size when all conditions including unstretched cells and cells stretched with V0 and V25 were considered. Using an extensive set of inhibitors, we also uncovered that the critical elements of the corresponding FDM pathway include cortical actin tension, intact microtubule network, proper mitochondrial fission and the ability of mitochondria to move along microtubules. Indeed, TMRM levels during V25 were reduced to those during V0 with the inhibition of non-muscle myosin II with blebbistatin, microtubule assembly with nocodazole, DRP1 (hence mitochondrial fission) with dynasore and motor activity with mitotic kinesin-like protein^24^. With regard to the reduced TMRM at V50, the increased level of DRP1 (Fig. 5D) likely contributed to the lowest ATP production (Fig. 6) and ATP level (Fig. 7C) via increased fission.

Despite the fragmentation at V50, a surprising finding was that TMRM levels went up following treatment with both H_2_O_2_ and AMP-PNP (Fig. 6). While the reason for this is not entirely clear, it is reminiscent of recent findings in pulmonary arterial hypertension^53^. Mitochondria of pulmonary VSMCs isolated from rats with pulmonary hypertension exhibited complex I-related dysfunction that lead to an unexpected glycolytic switch. Despite the enhanced glycolysis, mitochondrial ROS generation and mitochondrial membrane potential significantly increased^53^. This is in agreement with our results since the large amplitude fluctuations in stretch in V50 elevated mitochondrial ROS to a level 21 times higher than that during V25 (Fig. 7B) and both H_2_O_2_ and AMP-PNP increased cellular ROS relative to control conditions independent of stretch pattern (Fig. 8C). It is also notable that V50 also increased cellular ROS independent of H_2_O_2_ or AMP-PNP treatment (Fig. 8B). Since pulmonary arterial blood pressure displays spontaneous BPV^54^ that is amplified in pulmonary hypertension^55^, it is likely that in our study, mitochondrial ROS levels and high variability in V50 synergistically interacted to increase TMRM levels.

The primary physiological limitation of this study is that VSMCs were studied in culture without the native extracellular matrix. Hence the stiffness of the substrate including the flexible membrane was higher than that of the vascular wall. The biochemical and structural composition of the substrate was also different from those of the native vessel; for example, elastin and proteoglycans were not incorporated into the substrate. The cells may not have been fully confluent and cell shape and size can be altered by stretch. Previously, we reported that cell size decreases in V25 compared to V0, yet the latter has smaller cluster sizes^24^. The cells were only stretched for 4 hours which may also have affected mitochondrial organization and/or the biochemical results. Furthermore, VSMCs were not exposed to a static stretch corresponding to mean blood pressure and shear stress-induced endothelial cell signaling due to blood flow was not present. Despite all these limitations, our previous study showed that at least in one aspect, VSMC cell signaling related to contractility, the cell culture preparation is not a major limitation^24^.

A possible technical limitation is related to the small size of a single mitochondrion (~200 nm) because the structural data were derived from images that were obtained using a widefield microscope with a low axial resolution. To examine whether this affected our results, in a subset of cells cultured on glass we compared the exponents, cluster sizes and D_f_ from single slice z-stack images, 5-slice flattened images and widefield images. As can be seen in the Supplementary Figs. S2 and S3, flattening did not affect significantly the distributions and the exponent. Although the widefield imaging significantly shifted the cluster size distribution to the right, the exponents were virtually identical. This shift resulted in significantly larger cluster sizes from widefield images implying that cluster sizes depend on the absolute scale of imaging. More importantly, however, there was no change in the corresponding fractal dimension (Supplementary Fig. S4). Since the mitochondrial network form a self-similar fractal structure, the power law exponent and D_f_, both of which link structural features over multiple length scales, are invariant to resolution. As a consequence, the exponents and their changes with stretch pattern are not expected to be affected by the resolution of the widefield microscope and our conclusions regarding percolation remain valid. We also note that the percolation analysis is limited to clusters with appreciable inner membrane potential because the TMRM dye labels only the activated mitochondria. Unfortunately, we were not able to reliably quantify the exponents from MitoTracker Green-labeled mitochondria because the collagen coated elastic membranes exhibited a strong heterogeneous and diffuse background labeling (e.g., diffuse green in second image of top row, Fig. 5). To verify this, we also co-labeled mitochondria with TMRM and MitoTracker Green on glass substrate which eliminated the background and provided high quality images with 87% of TMRM-labeled pixels co-localized with MitoTracker Green pixels (Supplemental Fig. S5). Taken together, we conclude that FDM triggered percolation of both total and activated mitochondrial networks under V25 but not at V50. Although the activated mitochondria do not percolate at V0 and V6, this remains to be verified for the total mitochondria.

What are the implications of these results? There is evidence that an increase in BPV is an independent risk factor for various cardiovascular diseases and end-organ damage^56–61^. For example, patients with increased long-term variability in systolic BP have poor prognosis, despite greater use of add-on drugs^59^. Furthermore, patients with elevated beat-to-beat BPV (time scale of 1–30 min) or short-term BPV (time scale of 24 hours) also had a higher risk of target-organ damage^62^. On the other hand, normal BPV is significantly reduced by anesthesia^26^. Our results therefore suggest that pathological changes in BPV would generate different levels of variability in peak strains, which should significantly impact mitochondrial network structure with functional consequences. For example, the contractile apparatus of VSMCs is down-regulated by V0 compared to V25 which results in a reduced contractile force in vascular walls^24^. In the absence of natural BPV, the reduced VSMC contractility also substantially alters arterial wall ECM structure^63^. Alternatively, too much variability as in the case of V50, a fragmented mitochondrial network emerges with increased ROS production (Figs. 7 and 8). Since high ROS levels increase the contractile proteins in cells^64^ as well as inhibit force relaxation^65^, it is possible that the strong vascular contractility that underlies hypertension^66^ is also related to the increased BPV-driven ROS production.

In conclusion, mitochondrial cluster structure is tuned by FDM to ensure optimal electron transport chain efficiency including healthy ATP production with minimal ROS release. This optimal network architecture is dynamic and preserved during fusion and fission events at strain variability corresponding to normal BPV. It is likely that this optimal mechanical homeostasis is evolutionarily conserved. However, this dynamic state breaks down if cells experience pathologically low or high variability, oxidative stress or metabolic disturbance.

## Methods

All experiments were carried out using methods in accordance with relevant guidelines and regulations.

### Cell culture

Primary vascular smooth muscle cells (VMSCs) were isolated from bovine aortae obtained immediately after euthanasia from a local slaughter house (Research 87, Boylston, MA) and kept chilled until using the explant method^67,68^. After the adventitia was removed and the endothelial cells were scrapped off, the media layer of aorta was cut into small pieces which were then placed in a flask containing Dulbecco’s Modified Eagle Medium (DMEM-F12, Gibco) supplemented with 10% Fetal Bovine Serum (FBS, Gibco), penicillin and streptomycin (each 100 IU/ml) and 0.1 µg/µl primocin (InvivoGen). The samples were stored in a cell culture incubator at 5% CO_2_ and 37 °C. Cell outgrowth occurred within 7 days when the explants were removed. All experiments were carried out on first passage cells from six different isolations.

### Cyclic equi-biaxial stretch

Cells were plated on 6-well flexible membranes coated with type I collagen (Flexcell International Corp.). Before stretching, cells were incubated in DMEM without FBS for 24 h to arrest the cell cycle. One group of cells was stretched equi-biaxially using a sinusoidal stretch pattern with a peak surface area strain of 0.1 which we call monotonous stretch with 0% variability (V0). Three additional groups of cells received a stretch pattern with a cycle-by-cycle variability in surface area with the peak strain for each cycle pulled from a uniform distribution between A_min_ and A_max_ so that the mean was 0.1. Values for A_min_ were chosen to be 0.09375, 0.075 and 0.05 whereas those of A_max_ were 0.10625, 0.125 and 0.15. These ranges represent respectively 6.25% (V6), 25% (V25) and 50% (V50) cycle-by-cycle variability in peak strain around the mean of 0.1. In our previous study, we found that V25 represents variabilities in strain that corresponds to normal BPV in healthy subjects^24^. The increased strain variability in V50 was designed to mimic high BPV observed in hypertensive patients: when mean arterial pressure increased from about 90 mmHg to 140 mmHg, the standard deviation of half-hour mean blood pressures averaged over 24 h increased by a factor >2^69^. In contrast, V6 was chosen to represent reduced variability such as that during anesthesia. In children of ages between 5 and 13 years, total BPV computed in the frequency domain decreased by ~7 times under anesthesia compared to recovery^26^. Additionally, in order to maintain a constant strain rate, the frequency of stretching was inversely related to strain in each cycle such that the mean was at 1 Hz. All stretch patterns were applied for 4 h (n = 6) in a cell culture incubator at 5% CO_2_ and 37 °C.

### Mitochondrial membrane potential and ROS detection

The membrane potential was monitored using the dye tetramethylrhodamine methyl ester (TMRM). 45 min before the end of the 4 h stretching in the incubator, TMRM was added to the media (300 nM). The cellular ROS levels were determined using the 2′, 7′-dichlorodihydrofluorescein diacetate (H_2_DCFDA) fluorescence indicator dye at a concentration of 10 µM alone or together with the membrane potential indicator TMRM. Mitochondrial ROS was visualized using the MitoSOX Red mitochondrial superoxide indicator (1.7 μM) for live cell imaging. The MitoSOX Red dye was added to the wells 10 min before the end of the 4 h stretching in the incubator. In a set of additional experiments, both all and activated mitochondria were visualized by co-labeling VSMCs with MitoTracker Green (200 nM) and TMRM (300 nM) that were added to the wells 30 and 45 min before the end of the 4 h stretching, respectively. Furthermore, in another experiment, cells were plated on glass and co-labeled with TMRM and MitoTracker Green as above for subsequent imaging with a confocal microscope. All reagents were from Molecular Probes (ThermoFisher Scientific).

### Measurement of ATP content

The ATP content was measured using the ATPlite 1 step Luminescence ATP Detection Assay system (PerkinElmer) as described previously^24^. Briefly, at the last minute of the stretch protocol the media was replaced with the kit 1 step ATP stabilizer lyses reagent (300 μl/well) and cells were mixed for 2 minutes at 700 rpm on an orbital microplate shaker (Fisher Sci.). Cells were collected and added in 100 μl duplicates to a black microplate. Luminescence was read on a luminometer (Molecular Devices SpectraMax M5). The ATP content was corrected for the background reading and data were presented in normalized form.

### Measurement of mitochondrial proteins

Total proteins were extracted with cell lysis buffer (Sigma-Aldrich Inc.) in the presence of protease inhibitors (Halt™ Protease Inhibitor Cocktail (100×) and EDTA (100×), ThermoFisher Scientific) and quantified according to the BCA method (Pierce). Equal amounts (~3.8 µg) of protein/sample were separated on 4–20% gradient SDS-polyacrylamide gels (Bio-Rad Laboratories). Proteins were transferred to PVDF membranes (Bio-Rad Laboratories) and blocked with 5% bovine serum albumin (BSA) in PBS+ 0.05% Tween 20. Western blots were performed with mouse or rabbit antibodies for mitofusin-1 (1/2000, Abcam), mitofusin-2 (0.5 µg/ml, Abcam), DRP1 (1:500, Novus Biologicals), OPA1 (1:1000, Novus Biologicals), ATPB for the β subunit of the ATP synthase (0.5 µg/ml, Abcam) and VDAC1 (1 µg/ml, Abcam). To assure equal amount of loading, GAPDH (1 µg/ml, Abcam) was used as loading controls. Secondary antibodies were applied for 1 h and quantitative densitometry was performed after chemiluminescence detection using SuperSignal West Pico chemiluminescence substrate (Pierce) and corrections were made for background and loading control.

### Inhibitor and challenge studies

Cells were prepared as above and FBS withdrawal for 24 h was applied to bring cells to the same phase of the cell cycle. The same set of equi-biaxially stretch protocols was applied as for the control cells for 4 hours in the presence of the ATP analogue and ATPase inhibitor AMP-PNP (200 µM, Sigma-Aldrich) or hydrogen peroxide (H_2_O_2_, 50 µM, Sigma-Aldrich).

### Imaging and analysis

Immediately after the completion of the 4 h stretch, live cells were imaged using a Nikon Eclipse 50i microscope with a 60X water-immersion objective at a temperature ~35 °C in the presence of HEPES (Sigma-Aldrich) pH buffering. First, a few phase contrast images were captured followed by fluorescent imaging of TMRM and ROS (at least 50 cells per isolation). Single exposures of TMRM-labeled cells showed little to no photo bleaching with no difference in intensity-related morphometric indexes as in our previous study^24^. Before the semi-automated image processing, we carried out an extensive preliminary analysis to determine the best threshold to isolate mitochondrial clusters from the TMRM-labeled images. On each image, individual cells were manually segmented providing approximate cell areas for analysis of TMRM and ROS intensity. The background in both the TMRM and ROS images was determined as the mode of pixel values in a small area near each cell and subtracted from the cell image. Mean ROS intensity was then computed for each cell. The thresholded TMRM images served as a mask in each cell to compute the mean and maximum intensity per mitochondrial cluster and then the mean intensity per cell. From the binary TMRM images, we computed various statistics of cluster sizes including the mean, median, largest and second largest cluster and the variance per cell. To obtain the probability density distribution of cluster sizes, data from all cells were pulled. From the binary images, we also determined the fractal dimension D_f_ of mitochondria using the box counting algorithm. Briefly, a box of size *n* by *n* pixels was set up and the number of boxes (*N*) required to cover all mitochondria within a cell were determined. The procedure was then repeated for different values of *n* and D_f_ was obtained as the slope of the *N* vs *n* plot on a double logarithmic graph.

Since the size of a single mitochondrion is smaller than the resolution of the widefield Nikon Eclipse 50i microscope, in a subset of cell cultures, images were also acquired using a laser scanning confocal microscope (Olympus FV-3000) at an in-plane resolution of 0.173 μm and an axial optical resolution of 0.767 μm. Because of the design of the Flexcell plates, the samples on the elastic membrane could not be placed sufficiently close to the objective of the confocal microscope to allow focusing on the mitochondria. Therefore, cells were plated on glass substrate and co-labeled with TMRM and MitoTracker Green as above. The images were processed as above and the results were compared to the widefield images obtained with the Nikon microscope. Various structural features were computed from single slice images as well as from a flattened image using the “average intensity” z project option in ImageJ and compared to those obtained from the widefield microscopy. The co-labeling also allowed us to co-register the TMRM and MitoTracker images and determine the fraction of activated mitochondria in the cell. For the MitoTracker imaging, we applied a segmentation algorithm using the fibermetric built-in function of Matlab (The Mathworks, Inc., 2018) which identifies tubular structures using a Hessian-based multiscale filtering.

### Statistical analysis

Statistical data analysis was carried out in Sigmaplot (Systat Software, Inc., 2008) and Matlab (The Mathworks, Inc., 2018). Normally distributed data were compared using one-way or two-way ANOVA followed by Holm-Sidak post-hoc pairwise comparisons. Data that failed the normality or equal variance test, one-way ANOVA on ranks was applied followed by Dunn’s pairwise comparisons. For two-way ANOVA, the data were transformed before the parametric test. In cases where we did not find a proper transformation, we carried out a series of one-way ANOVA on ranks examining each factor separately. For normally distributed data we plotted the mean and SD (Fig. 4) while data following other distributions, were represented by box plots as follows: the boundary of the box closest to zero marks the 25th percentile, the line within the box is the median, the boundary of the box farthest from zero is the 75th percentile and the error bars above and below the box indicate the 90th and 10th percentiles. These graphs also show the outlying points (e.g., Fig. 4B). Exponents were assumed to have normal distributions and the values of the mean and SD obtained from linear regression on double logarithmic plots were compared using one-way ANOVA. Distributions were compared using K-S tests. Significance was accepted at the level of p < 0.05.

## Supplementary information

Supplementary Information.
