# Supplementary material for: Tuning mitochondrial structure and function to criticality by fluctuation-driven mechanotransduction
Source: Sci Rep. 2020 Jan 15;10:407. doi: 10.1038/s41598-019-57301-1 (PMC6962425; doi:10.1038/s41598-019-57301-1)
Supplement: Supplementary file 1 — Supplementary Information. [file 41598_2019_57301_MOESM1_ESM.docx]

Title

- Tuning mitochondrial structure and function to criticality by fluctuation-driven mechanotransduction
- Supplementary Figures

**Authors**

Erzsébet Bartolák-Suki, Béla Suki*

**Affiliations**

Department of Biomedical Engineering,

44 Cummington Mall

Boston University, Boston, MA 02215

Tel: 1 617 353-5907

Email: [bsuki@bu.edu](mailto:bsuki@bu.edu)

Supplementary Figures


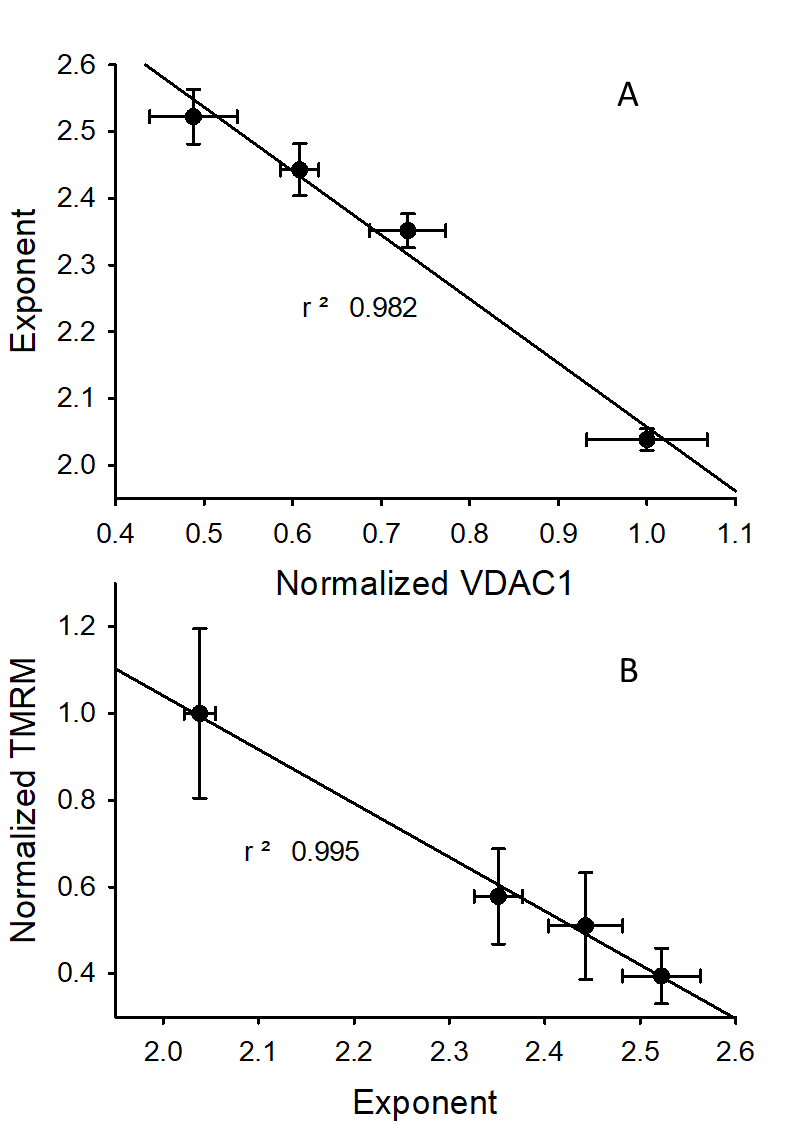


**Fig. S1. Correlation plots between biochemical, structural and functional properties of the mitochondria.** (A) Regression between the normalized values of VDAC1 from Fig. 5E and the exponent of the power law distributions from Fig. 2D. (B) Regression between the exponent of the power law distributions and the normalized TMRM intensity from Fig. 6. Data from all stretch groups are included.


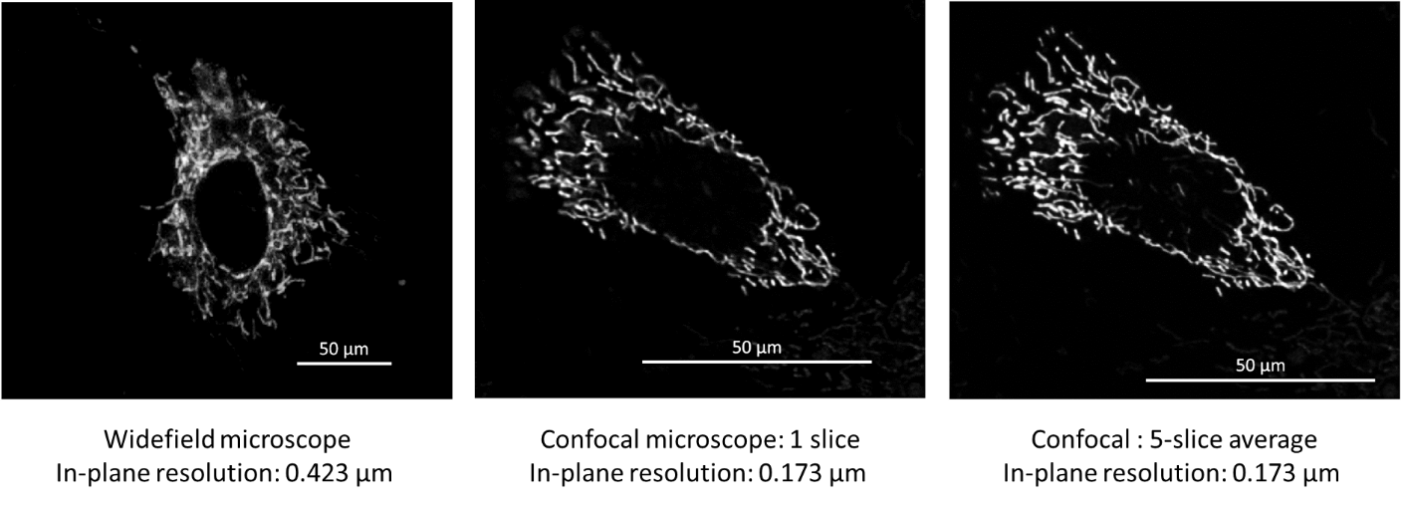


**Fig. S2. Example images of TMRM-labeled mitochondria in vascular smooth muscle cells obtained with three different methods.** Vascular smooth muscle cells were prepared as described in the main text and plated on glass slides. Cells were imaged using an upright Nikon Eclipse 50i widefield microscope with a 60X water-immersion objective (left panel) and with an inverted Olympus FV 3000 confocal microscope (middle and right panels). A single image per cell was obtained with the widefield microscope whereas a z-stack per cell was collected at a true optical resolution of 0.767 μm in the z direction with the confocal microscope. A 3-step iterative maximum likelihood deconvolution was also applied to all confocal images. The middle image was selected to be the one having the most number of mitochondrial clusters. The image on the right was then computed as the average of 5 single slice images centered around the image in the middle panel. The average image appears to contain a denser mitochondrial network. Notice also the higher in-plane resolution and the sharper details on the confocal images compared to the image on the left panel.

**Fig. S3. The probability density distribution of mitochondrial cluster sizes and the corresponding exponents.** A set of 25 images were collected with the wide field upright microscope (e.g. Fig. S2 left panel) and a set of 10 z-stacks of individual cells were obtained with the inverted confocal microscope (e.g. Fig. S2 middle and right panels). Each image was first processed as described in the Methods to obtain a set of clusters from which the distributions were computed. The distributions showed a long tail on the double logarithmic graph and their negative slope α was taken as the exponent of each power law distribution. The distributions from the single slices (red line) and the 5-slice average images (blue line) were nearly identical except that the latter included larger clusters. The distribution from the wide field upright microscope (green line) was shifted to the right. The black line show the fit of a straight line to the widefield microscope data. The values of the exponent α were very similar and there was no statistically significant difference among the 3 cases.


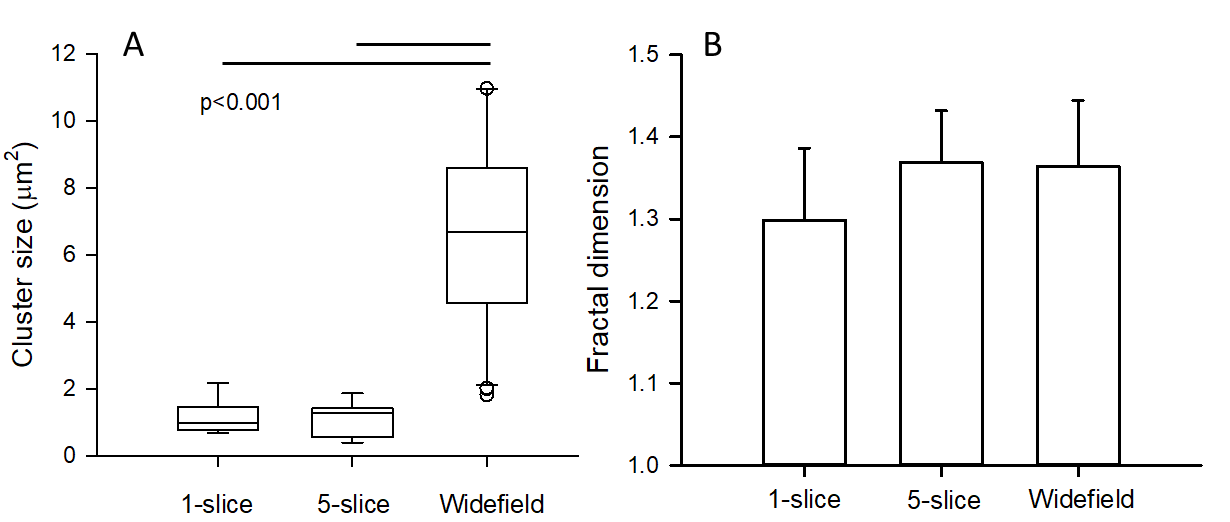


**Fig. S4. Analysis of cluster structure with three methods.** Median cluster sizes (A) and the fractal dimensions (B) corresponding to images as in Supplementary Fig. S2 and the distributions in Supplementary Fig. S3. The cluster sizes from the single slice and the 5-slice averaged images were in in good agreement (no statistical difference). However, the median cluster size from the wide field upright microscope was statistically significantly higher than the median cluster sizes from the confocal images in agreement with the shift of the distribution to the right in Supplementary Fig. S3. This is a result of the thicker focal plane in the widefield microscope. There was no difference in the fractal dimensions between any of the imaging cases.

A


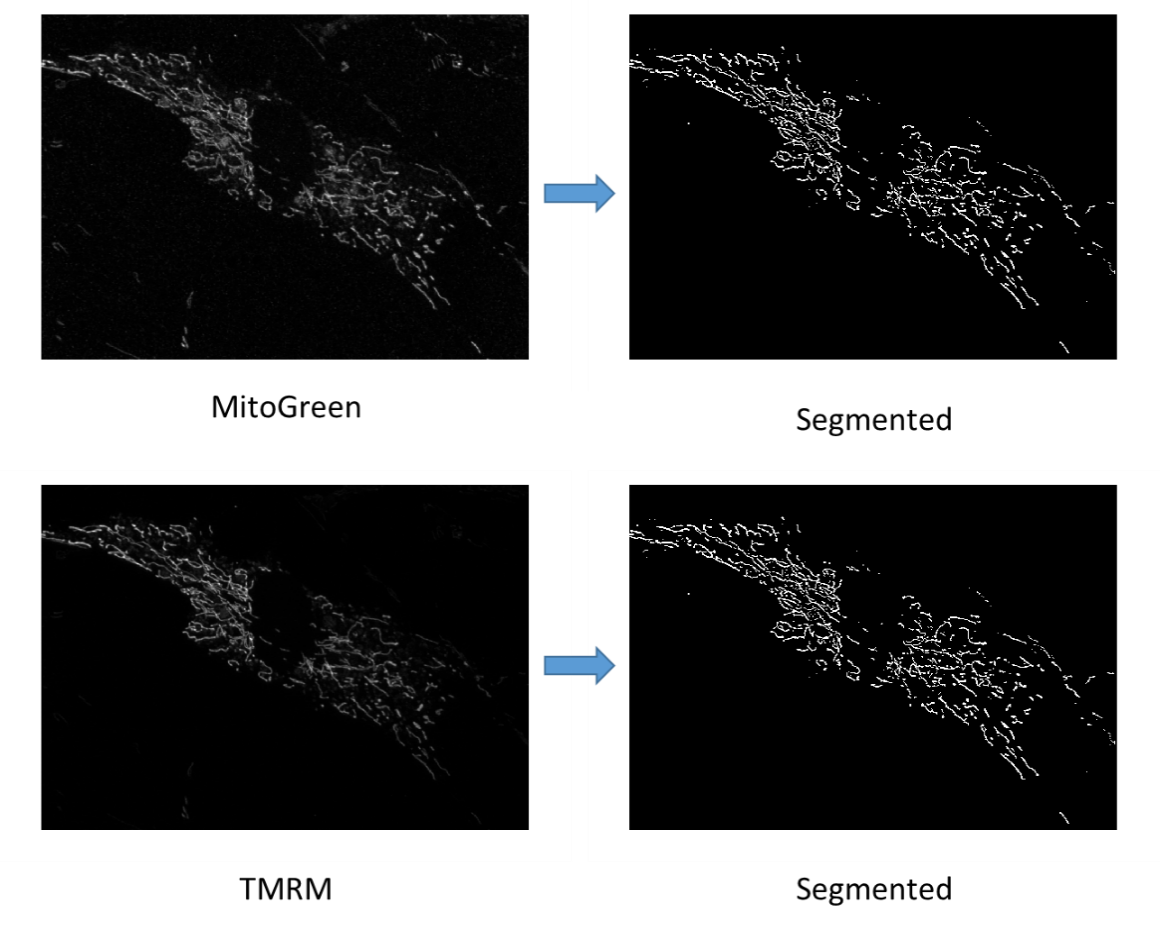


B

**Fig. S5. High resolution imaging of active and all mitochondria.** A) Examples of TMRM (top left) and MitoTracker Green (bottom left) from single slice confocal images and their corresponding segmented binary images (top right and bottom right, respectively). VSMC were cultured on glass and imaged as in Fig. S2 middle. B) Co-localization of TMRM as an indicator of active mitochondria and MitoTracker Green as an indicator of all mitochondria: red: only TMRM; green: only MitoTracker Green; blue: co-localized TMRM and MitoTracker Green. The red image was co-registered to the green. Notice that some red pixels are seen alone (~13% of all red) which is an artifact likely because the MitoTracker Green did not label those regions strong enough and following image processing the green disappeared. It is also noteworthy that the blue pixels take up ~61% of the green pixels suggesting that 61% of the mitochondria are actively charged.
